# Comparative analysis of gene expression patterns in the HTLV-1 infected T-cell clones

**DOI:** 10.1186/1742-4690-12-S1-P14

**Published:** 2015-08-28

**Authors:** Anat Melamed, Hiroko Yaguchi, Aileen Rowan, Lucy Cook, Charles R M Bangham

**Affiliations:** 1 Imperial College London, London UK

## 

The integration of Human T-lymphotropic virus type I (HTLV-1) is known to be diverse but biased towards genomically active regions. Each infected clone is identified by a unique integration site position. The integration site position is known to be associated with the expression pattern of the provirus and its propensity to expand in vivo but it is not known how it would impact the gene expression of the host genes. The viral transactivator Tax has been shown (mostly in vitro) to be capable of altering the expression of many host genes however it is not known what role the genomic integration site may have in determining the alteration of host gene expression or how the gene expression pattern would vary from one infected T-cell clone to another. We have developed a panel of HTLV-1 infected T-cell clones. CD4+ T-cells were isolated from HTLV-1 infected individuals and cloned by limiting dilution. Each clone was cultured in the presence of integration inhibitor to prevent super infection and defined by a single unique integration site. Uninfected T cell clones were also established in the same way. In order to address this question and to analyze gene expression pattern, total RNAs were isolated from each clone for RNA sequencing (RNA-seq). We will present the results of high-throughput sequencing of the human and viral mRNA, highlighting the similar and variable between the different clones.

# Poster award winnder - 2nd place

